# Technogenic contamination of livestock- and fish-derived food products with heavy metals and radionuclides in industrial and post-nuclear regions of Kazakhstan

**DOI:** 10.14202/vetworld.2026.1052-1068

**Published:** 2026-03-15

**Authors:** Zhanat Adilbekov, Raikhan Mustafina, Shyngys Suleimenov, Ainur Serikova, Gulnur Zhuzzassarova, Zhanbolat Suranshiyev, Aslan Bainiyazov

**Affiliations:** 1 Group of Educational Programs “Veterinary”, Institute of Animal Science and Veterinary Medicine, NJSC “S. Seifullin Kazakh Agro Technical Research University”, Astana, Kazakhstan; 2 Department of Veterinary, Research School of Veterinary Medicine and Agriculture, NJSC “Shakarim University”, Semey, Kazakhstan

**Keywords:** animal-derived foods, food safety, heavy metals, Kazakhstan, livestock products, radionuclides, technogenic contamination, toxic elements

## Abstract

**Background and Aim:**

Technogenic pollution from industrial activities and legacy nuclear testing remains a major environmental concern in several regions of Kazakhstan. Contaminants such as heavy metals and radionuclides can migrate through environmental matrixes into the food chain, potentially compromising the safety of livestock- and fish-derived food products. This study aimed to assess contamination levels of toxic elements and radionuclides in meat, poultry, milk, dairy products, and freshwater fish from technogenically hazardous regions of Eastern, Northern, and Central Kazakhstan and to evaluate their compliance with established safety standards.

**Materials and Methods:**

A cross-sectional monitoring study was conducted from August 2024 to September 2025 in the Abai, Akmola, and Karaganda regions. A total of 383 samples of meat, milk, and dairy products and 143 freshwater fish samples were collected from farms, retail markets, and local water bodies. Concentrations of lead (Pb), cadmium (Cd), copper (Cu), and zinc (Zn) were determined using inductively coupled plasma optical emission spectrometry. Radionuclides cesium-137 and strontium-90 were analyzed using a multichannel gamma spectrometer with radiochemical methods where applicable. Descriptive statistics and analysis of variance were performed using IBM SPSS Statistics version 25, with p < 0.05 considered statistically significant.

**Results:**

Elevated concentrations of toxic elements were detected in several livestock products. The Karaganda region showed the highest contamination, including exceedances of Pb, Cd, Cu, and Zn in horse meat and elevated Cu in beef. In the Akmola region, exceedances were mainly observed for Cu and occasionally Cd, whereas in the Abai region Cd exceedances predominated. Poultry meat generally met safety standards, except for Cd exceedance in chicken from the Akmola region. In dairy products, Cd and Cu exceeded permissible levels in cottage cheese and milk in selected districts, while Pb exceeded limits in whole milk samples from the Abai region. Freshwater fish contained detectable heavy metals, but concentrations remained below maximum permissible limits. Radionuclide levels in all tested products were substantially below regulatory thresholds, and no statistically significant regional differences were observed.

**Conclusion:**

The findings demonstrate localized accumulation of toxic elements in livestock-derived foods in industrially impacted regions of Kazakhstan, while radionuclide contamination remains within safe limits. Continuous environmental monitoring and strengthened food safety surveillance are required to minimize health risks and ensure the safety of animal-derived food products in technogenically affected areas.

## INTRODUCTION

Under conditions of intensive industrial development, the concentrations of technogenic pollutants in soil, water resources, atmospheric air, and feed in many regions significantly exceed maximum permissible concentrations (MPCs). Major pollution sources include metallurgical, chemical, fuel and energy, biotechnological, and processing industries. Exposure to these factors results in metabolic maladaptation in animals, manifested by pronounced metabolic disturbances, reduced productivity and reproductive performance, and deterioration in the nutritional and biological value of livestock products [1–6].

In recent years, considerable attention has been devoted to investigating the pathways through which technogenic pollutants enter environmental matrixes, including food products, as well as to developing preventive measures aimed at reducing their adverse effects on human health. Contemporary studies confirm that chemical contaminants enter the food chain through contaminated soils, water resources, atmospheric emissions, and feed, thereby creating persistent ecotoxicological risks [7–9]. Heavy metals such as lead (Pb), cadmium (Cd), mercury (Hg), and arsenic (As) actively migrate along the biogeochemical chain “soil–plant–animal–food product,” accumulating in animal tissues and organs and posing a threat to public health [10, 11].

These findings highlight the need to develop modern monitoring systems, preventive approaches, and risk management strategies aimed at minimizing the transfer of technogenic pollutants into food products and ensuring population safety [12, 13].

Despite Kazakhstan’s vast steppe ecosystems and traditionally low population density, intensified industrial activity and urbanization have led to increasing soil contamination with heavy metals. Recent studies have identified localized contamination hotspots, emphasizing growing environmental risks and potential threats to public health associated with toxic element accumulation in environmental matrixes [14].

Elevated concentrations of Pb, Cd, copper (Cu), zinc (Zn), and chromium (Cr) were recorded in urban environments during national soil monitoring conducted between 2010 and 2018, with the highest levels observed in Balkhash, Ust-Kamenogorsk, Ridder, and Shymkent. Average Pb and Cd values in these regions exceeded geoaccumulation thresholds, indicating substantial anthropogenic influence and elevated environmental risk. Probabilistic risk assessments demonstrated potential exposure of both adults and children to carcinogenic levels of heavy metals [15].

Certain territories of Northern, Central, and Eastern Kazakhstan have experienced substantial technogenic pressure in recent decades. In the Akmola region, major environmental pollution sources include enterprises engaged in the extraction and processing of gold-bearing ores, uranium mining operations, facilities using heap-leaching technology, and uranium ore mining and processing complexes where open-pit extraction methods were previously applied [16–19].

In the Karaganda region, more than 400 enterprises exert negative environmental impacts. The ecological situation in this region is considered unfavorable due to the high concentration of ferrous and non-ferrous metallurgy, power-generation facilities, and other industrial installations [20]. A model-based assessment demonstrated that operations of the metallurgical plant in Temirtau between 1996 and 2023 substantially contributed to air pollution and associated population health risks [21]. Persistently elevated concentrations of heavy metals (As, Pb, Cd, Zn, Cu, and Cr) have been reported in soils and water bodies within industrially affected areas of the Karaganda region [22, 23]. Investigations of Pb and petroleum products in cities across the region also confirm the anthropogenic origin of pollution and its spatial association with coal-mining and metallurgical enterprises [24, 25].

Significant contamination of certain water bodies in Central Kazakhstan has also been documented, including the Nura, Kengir, Ishim, and Tobol rivers, as well as Lake Balkhash, which receive wastewater containing substances harmful to aquatic organisms. Industrial enterprises in Karaganda and Temirtau annually discharge wastewater containing ash and slaked lime, waste products from synthetic rubber production and other industries, into the Nura River and the Karaganda Reservoir. The Nura River is additionally contaminated with mercury. Another major environmental concern for Lake Balkhash is industrial waste from the Balkhashtsvetmet plant, whose emissions contain sulfur dioxide as well as multiple heavy metals [26, 27].

An additional environmental stress factor is the former Semipalatinsk Nuclear Test Site (SNTS) [28, 29]. Despite the closure of the test site, its medical, social, and environmental consequences remain relevant and require comprehensive long-term investigation and mitigation across generations [30–34]. Radionuclides participate actively in biogeochemical cycles and may enter human and animal organisms through the food chain: atmosphere → water → soil → plant → milk, meat, and fish products → human [35, 36].

Although numerous studies have documented environmental contamination of soils, water bodies, and atmospheric media in industrial and post-nuclear regions of Kazakhstan, available evidence primarily focuses on environmental matrixes rather than on the transfer of contaminants into animal-derived food products. Data describing the accumulation of heavy metals and radionuclides along the “environment → feed → animal → food product” pathway remain fragmented, geographically limited, and often restricted to single product types or individual contaminants. In particular, comparative assessments integrating multiple livestock products, dairy products, and freshwater fish across regions exposed to different technogenic pressures, such as mining-industrial zones and territories adjacent to the SNTS, are scarce. Moreover, recent standardized monitoring data evaluating compliance of animal-derived foods with current regulatory MPCs are limited, hindering accurate risk assessment for human consumption and the development of evidence-based food safety strategies. Therefore, comprehensive regionally stratified studies combining multi-product sampling with simultaneous evaluation of toxic elements and radionuclides are needed to better characterize contamination patterns and support national surveillance and public health protection.

Therefore, the present study aimed to assess the degree of contamination of livestock- and fish-derived food products with toxic elements and radionuclides in technogenically hazardous regions of Eastern, Northern, and Central Kazakhstan. Specifically, the objectives were: (i) to quantify residual levels of Pb, Cd, Cu, Zn, and selected radionuclides in meat from major farm animal species and poultry, in milk and dairy products, and in freshwater fish from local water bodies; (ii) to evaluate whether the detected concentrations comply with established MPCs; (iii) to compare contamination patterns among the Abai, Akmola, and Karaganda regions characterized by differing industrial and post-nuclear environmental pressures; and (iv) to identify potential environmental pathways contributing to contaminant transfer into animal-derived foods. By providing integrated empirical data from multiple product categories and regions, this study seeks to strengthen the evidence base for environmental-food chain monitoring and contribute to improving food safety management in Kazakhstan’s technogenically impacted territories.

## MATERIALS AND METHODS

### Ethical approval

Ethical approval was not required for this study because it did not involve experimental procedures on live animals. The research was based exclusively on the collection and laboratory analysis of livestock- and fish-derived food products obtained from farms, retail markets, and natural water bodies as part of a cross-sectional environmental monitoring survey. No animals were subjected to handling, treatment, or invasive procedures specifically for research purposes.

All sampling procedures were conducted in accordance with the Rules for Sampling of Transported (Carried) Objects and Biological Material approved by the Order of the Minister of Agriculture of the Republic of Kazakhstan dated 9 July 2015 No. 11618 (as amended on 6 April 2020). The study complied with national regulations governing food safety, laboratory analysis, and environmental monitoring under Technical Regulation of the Customs Union TR CU 021/2011 “On Food Safety.”

### Study period and location

This cross-sectional monitoring study was conducted in three regions of Kazakhstan from August 2024 to September 2025, focusing on areas exposed to the highest technogenic impact.

Sampling locations were selected based on the intensity of technogenic impact on the regions, including active sources of environmental pollution, particularly facilities related to ferrous and non-ferrous metallurgy, the energy sector, uranium mining, gold processing, and areas affected by nuclear testing.

Monitoring sites for sampling meat, milk, and dairy products were established in the Abai, Akmola, and Karaganda regions. The Abai region is located in Eastern Kazakhstan and covers approximately 185,500 km² (the territory includes the former SNTS). The Akmola region, located in Northern Kazakhstan, has an area of 146,200 km² and is characterized by a high concentration of industrial facilities, including gold and uranium mining and processing enterprises and chemical industry plants. The Karaganda region is the largest industrial region in Central Kazakhstan, with a total area of 239,045 km².

### Sample size

The main types of locally produced meat (beef, horse meat, and mutton), poultry (chicken, goose, and duck meat), and dairy products (whole milk and cottage cheese) were selected for analysis.

At least six samples of each product type were collected for statistical reliability. A total of 384 samples of meat and dairy products and 144 fish samples were obtained.

The sample size was based on previously published studies on toxic elements and radionuclides in animal products. A minimum of six independent samples per product type and sampling site was considered sufficient for descriptive statistical analysis. An a priori power calculation (G*Power) was not applied because the study was designed as a cross-sectional descriptive monitoring survey rather than a hypothesis-driven experimental study.

### Sample selection criteria

Samples were purposively collected from areas with the highest technogenic load, including territories hosting uranium- and gold-ore mining and processing plants, facilities applying heap-leaching technology, areas with a high density of ferrous and non-ferrous metallurgical industries, and the former SNTS. Repeated sampling from the same source was avoided.

### Sample collection procedures

Samples were collected in accordance with the Rules for Sampling of Transported (Carried) Objects and Biological Material, approved by the Order of the Minister of Agriculture of the Republic of Kazakhstan dated 9 July 2015 No. 11618 (as amended on 6 April 2020).

Samples were collected from three districts (Abai, Aksuat, and Ayagoz) in the Abai region. Sampling was conducted in the Zerendinsky, Akkol, and Bulandy districts in the Akmola region and in the Abai, Bukhar-Zhyrau, and Aktogay districts in the Karaganda region. Sampling was performed at farms and food market stalls in the cities of Astana, Karaganda, Kokshetau, and Temirtau. Freshwater fish samples were collected from local water bodies in the Akmola and Karaganda regions.

### Meat sampling

Meat subsamples were collected from carcasses or half-carcasses as pieces weighing at least 200 g from one of the following locations: the slaughter cut area, the shoulder region, or the thigh region (from thick muscle portions). Point samples were combined into a composite sample, from which a mean sample weighing at least 1 kg was prepared.

### Poultry sampling

Chicken and duck samples were collected as whole carcasses, whereas geese were sampled as quarter carcasses. Poultry carcasses were randomly selected from the batches supplied for retail sale. Point samples were combined into a composite sample, from which a 1 kg mean sample was prepared.

### Fish sampling

Fish were collected from different parts of each batch. Approximately 1%–2% of fish were selected based on weight: for fish weighing up to 100 g, 5–7 individuals were sampled; for fish weighing up to 1 kg, 1–2 individuals were sampled. A mean sample weighing 1 kg was prepared.

### Milk and dairy sampling

Before sampling milk from cans, the milk was thoroughly mixed using a measuring cup. A 1 L sample was collected from the composite sample. For homemade cottage cheese and brined cheese, 200 g of point samples were taken from different layers of each product, and a 1 kg mean sample was prepared.

### Storage and transport

Each sample was placed in a sterile plastic bag (milk samples were collected in plastic bottles), labeled with a unique code, stored at ≤4°C, and delivered to the laboratory in insulated containers within 24 h. During storage, the collected samples were frozen at −20°C.

### Laboratory analysis

#### Determination of toxic elements

Toxic elements in the collected samples were determined using an EXPEC 6500 instrument (Focused Photonics (Hangzhou) Inc., Hangzhou, China) and an inductively coupled plasma optical emission spectrometer (ICP-OES), which is registered in the State Register of Measuring Instruments under No. 86919-22. Radionuclide determinations in samples from the study areas were performed using a multichannel Canberra gamma spectrometer (Mirion Technologies Canberra, Inc., Meriden, CT, USA).

#### Sample preparation

Samples were thawed at +2°C to +6°C, and inedible components (large bones/cartilage) were removed along with excess surface fat when necessary. The material was cut into 1–2 cm pieces and homogenized using a meat grinder. The resulting mass was mixed for 3–5 min until complete homogeneity was achieved.

The crucible was removed and cooled to room temperature during mineralization. The ash was moistened with 1 mL of concentrated nitric acid (HNO_3_). The acid was evaporated to dryness on a hotplate under gentle heating. Subsequently, the crucible was placed back into a muffle furnace at 250°C, and the temperature was increased to 450°C and maintained for 1 h. Mineralization was considered complete when the ash became white (moist salts) with no charred particles. The moist salts were dissolved in 1 mL of HCl, and the solution was quantitatively transferred to a 25 mL volumetric flask and brought to volume with the background electrolyte solution.

#### Preparation of milk and dairy products

Milk was thoroughly mixed in the original container before sampling. The milk was mixed again before pouring into the measuring vessel while avoiding foam formation. An aliquot of 20 mL of the thoroughly mixed milk sample was transferred into a porcelain dish and dried in a drying oven with a gradual increase in temperature to 150°C until complete drying. The dried sample was then charred on a sand bath or hot plate until smoke emission ceased.

The dish containing the charred sample was placed in a muffle furnace at approximately 250°C. The furnace temperature was gradually increased by 50°C every 30 min until it reached 450°C. Mineralization was continued at 450°C until the formation of gray ash. The ash-containing dish was removed and allowed to cool to 20°C–23°C. The ash was moistened with 1 mL of concentrated nitric acid (HNO3). The acid was evaporated to dryness on a hotplate under gentle heating, and the dish was returned to the muffle furnace at 250°C. The temperature was gradually increased to 450°C and maintained for 1 h. Mineralization was considered complete when the ash became white (moist salts) with no charred particles. The moist salts were dissolved in 1 mL of HCl, and the solution was quantitatively transferred into a 25 mL volumetric flask and brought to volume with the background electrolyte solution.

#### Determination of toxic elements by ICP-OES

Heavy metals were determined using the EXPEC 6500 spectrometer (Focused Photonics (Hangzhou) Inc.) based on ICP-OES. The analysis was based on optical emission spectrometry, in which the sample is atomized in a plasma torch and the excited atoms emit element-specific radiation. The instrument detector detects the emitted light, allowing simultaneous determination of multiple elements with high sensitivity.

Instrument parameters were as follows: spectral range 160–900 nm; dispersion system echelle (2D) with no moving parts; optical resolution (FWHM) ≤7 pm at 200 nm; optical chamber temperature stabilization 36°C ± 0.1°C; and argon purging of the optical path for the ultraviolet region.

#### Detection limits

The limit of detection (LOD) and limit of quantification (LOQ) for Pb, Cd, Cu, and Zn were defined as follows: LOD was assumed equal to the method detection limit (MDL), and LOQ was calculated as the minimum level (ML) using the formula ML = 3.18 × MDL.

The obtained LOD and LOQ values were as follows: Pb 0.010 and 0.0318 mg/L; Cd 0.001 and 0.00318 mg/L; Cu 0.003 and 0.00954 mg/L; Zn 0.002 and 0.00636 mg/L.

For solid samples (mass-based conversion), the LOD and LOQ values were 2.0 and 6.36 mg/kg for Pb, 0.2 and 0.636 mg/kg for Cd, 0.5 and 1.59 mg/kg for Cu, and 0.3 and 0.954 mg/kg for Zn, respectively.

#### Determination of radionuclides

A Canberra gamma spectrometer (Mirion Technologies Canberra, Inc.) equipped with a GC 2019 detector and a DSA-1000 multichannel analyzer was used to identify and quantitatively determine radionuclides in various samples. The system is based on the detection of gamma photons by a high-purity germanium detector and their conversion into electrical signals, which are processed to generate an energy spectrum. Peaks in the spectrum correspond to the energies and activities of the measured isotopes. The GC 2019 detector is equipped with thermoelectric cooling, eliminating the need for a liquid nitrogen cryostat.

The radionuclides analyzed included cesium-137 (137 Cs) and strontium-90. Determination of strontium-90 was performed using a radiochemical method in accordance with GOST R 54017-2010 and MG 2.6.1.2398-08. The ash was dissolved in mineral acid, followed by radiochemical separation of strontium. Prior to activity measurement, samples were aged until radioactive equilibrium in the strontium-90–yttrium-90 system was established. The detection limit was 1–3 Bq/kg.

#### Quality assurance and quality control (QA/QC)

A control standard or source was used at the beginning and end of each measurement batch to verify the stability of system performance (peak positions and sensitivity). A laboratory control sample was prepared by adding a known activity of the target radionuclide to a model solution or clean matrix to confirm method accuracy, followed by full sample preparation and measurement.

For each batch, a reagent blank was included and measured alongside the samples to control for contamination from reagents and labware, detect cross-contamination, account for background contributions, and calculate the minimum detectable activity. A second aliquot (duplicate) was processed independently for selected samples to assess repeatability and stability of radiochemical separation and measurement.

The acceptance criteria were as follows: recovery for laboratory control samples 70%–130%; chemical yield for strontium-90 radiochemistry 60%–90% (with an acceptance threshold not lower than 50%); and tracer-based recovery (if applied) 70%–110%.

#### Instrument performance control

Daily performance control before each measurement series was conducted using a certified multi-energy reference source (typically europium-152 or a set of 137Cs/cobalt-60/barium-133). Peak positions were checked against known energies (e.g., 661.7 keV for 137 Cs). The acceptance criterion was defined in the standard operating procedure (typically a peak shift of no more than ±0.5 keV or ±1 channel across the full energy range). If this criterion was exceeded, energy recalibration was performed, followed by repeated verification.

Background spectra were regularly recorded with the chamber closed (without a sample) and compared with baseline values. The criterion was that background levels should not increase relative to the reference baseline; in the case of elevated background, shielding integrity, chamber cleanliness, and potential nearby contamination sources were checked.

#### Regulatory standards

In Kazakhstan, MPCs of toxic elements and radionuclides in food products are regulated by Technical Regulation of the Customs Union TR CU 021/2011 “On Food Safety,” approved by Decision of the Customs Union Commission dated 9 December 2011, No. 880 (as amended on 10 June 2014).

### Statistical analysis

Data processing and statistical analysis were performed using IBM SPSS Statistics for Social Sciences version 25.0 (IBM Corp., Armonk, NY, USA). Descriptive statistics (mean, standard deviation, minimum, and maximum) were calculated. The Shapiro–Wilk test was used to assess the normality of the data distribution. Group comparisons were performed using one-way analysis of variance, and statistical significance was set at p < 0.05.

## RESULTS

Monitoring was conducted to assess contamination of meat from various livestock species and poultry, dairy products, and freshwater fish with heavy metals (Pb, Cd, Cu, and Zn) in selected regions of Northern, Central, and Eastern Kazakhstan located in areas experiencing the highest technogenic burden.

### Meat samples from various animal species

Table 1 presents the results of the analysis of meat samples from different animal species. As shown in the Table 1, the meat samples from the Akmola region exhibited a slight exceedance of the MPCs for Cd in beef samples from the Zerenda district at a concentration of 0.068 ± 0.002 mg/kg, from the Shortandy district at 0.071 ± 0.008 mg/kg, and from the Akkol district at 0.087 ± 0.006 mg/kg (MPCs 0.05 mg/kg), while in horse meat samples from the Shortandy district it reached 0.12 ± 0.04 mg/kg, which exceeds the MPCs by 2.4 times. A high Cu content was detected in beef samples from the Zerenda district at 14.5 ± 2.32 mg/kg (almost 3 times higher than the norm), in horse meat at 23.0 ± 2.21 mg/kg (4.6 times higher than the norm), and in samples from the Akkol district in lamb at 9.0 ± 0.21 mg/kg (1.8 times higher than the norm), in horse meat at 8.3 ± 0.21 mg/kg (1.6 times), and in beef at 9.45 ± 1.32 mg/kg (1.9 times higher than the norm).

**Table 1:** Content of toxic elements in farm animal meat.

Regions	Type of meat	Lead (mg/kg)	Cadmium (mg/kg)	Copper (mg/kg)	Zinc (mg/kg)
Akmola region					
Zerenda	Beef, n = 8	0.031 ± 0.008	0.068 ± 0.0021	14.5 ± 2.32	0.29 ± 0.027
	Horse meat, n = 6	0.0024 ± 0.0006	n/d	23.0 ± 2.21	1.77 ± 0.26
	Lamb meat, n = 6	n/d	n/d	1.0 ± 0.27	26.80 ± 2.31
Akkol	Beef, n = 8	0.065 ± 0.008	0.087 ± 0.006	9.45 ± 1.32	0.0021 ± 0.0007
	Horse meat, n = 6	0.0023 ± 0.0006	n/d	8.3 ± 0.21	1.77 ± 0.26
	Lamb meat, n = 6	n/d	n/d	9.0 ± 1.23	3.3 ± 0.64
Shortandy	Beef, n = 8	0.088 ± 0.006	0.071 ± 0.008	1.3 ± 0.4	11.2 ± 0.85
	Horse meat, n = 6	0.09 ± 0.029	0.12 ± 0.04	0.013 ± 0.005	n/d
	Lamb meat, n = 6	0.00029 ± 0.0001	0.0014 ± 0.0005	n/d	0.18 ± 0.07
Karaganda region					
Bukhar-Zhyrau	Beef, n = 6	n/d	n/d	5.07 ± 0.024	4.3 ± 0.6
	Horse meat, n = 6	0.71 ± 0.0013	0.216 ± 2.26	0.031 ± 0.002	36.6 ± 4.62
	Lamb meat, n = 6	1.11 ± 0.046	0.11 ± 0.64	1.84 ± 0.16	8.73 ± 2.72
Abai	Beef, n = 6	0.0037 ± 0.00016	n/d	0.012 ± 0.0002	n/d
	Horse meat, n = 6	n/d	0.011 ± 0.00012	0.497 ± 0.0002	53.7 ± 0.62
	Lamb meat, n = 6	0.00061 ± 0.0000	0.016 ± 0.0024	0.052 ± 0.00017	n/d
Aktogay	Beef, n = 6	0.067 ± 0.00012	0.0056 ± 0.0002	52.95 ± 8.26	2.6 ± 0.032
	Horse meat, n = 6	1.94 ± 0.0024	0.0112 ± 0.0012	0.055 ± 0.0022	155.3 ± 2.26
	Lamb meat, n = 6	n/d	n/d	0.026 ± 0.00018	7.1 ± 0.00013
Abai region (formerly the East Kazakhstan region)					
Abai	Beef, n = 6	0.20 ± 0.04	0.135 ± 0.035	2.000 ± 0.300	4.24 ± 0.37
	Horse meat, n = 6	0.175 ± 0.02	0.100 ± 0.075	1.850 ± 0.060	4.30 ± 0.55
	Lamb meat, n = 6	0.225 ± 0.04	0.140 ± 0.035	1.800 ± 0.030	4.25 ± 0.30
Ayagoz	Beef, n = 6	0.100 ± 0.03	0.040 ± 0.020	1.300 ± 0.020	3.72 ± 0.20
	Horse meat, n = 6	0.105 ± 0.02	0.105 ± 0.015	1.550 ± 0.020	3.85 ± 0.25
	Lamb meat, n = 6	0.105 ± 0.01	0.050 ± 0.056	1.450 ± 0.020	4.11 ± 0.35
Aksuat	Beef, n = 6	0.085 ± 0.01	0.025 ± 0.025	1.250 ± 0.015	3.60 ± 0.15
	Horse meat, n = 6	0.230 ± 1.57	0.020 ± 0.170	1.100 ± 0.010	3.40 ± 0.20
	Lamb meat, n = 6	0.080 ± 1.32	0.035 ± 0.030	1.200 ± 0.025	3.40 ± 0.10
MPCs (TR CU 021/2011)		0.5	0.05	5.0	70

Note: n/d, not detected.TR CU 021/2011, Technical Regulation of the Customs Union 021/2011.

During the study of meat samples from the Karaganda region, a slight exceedance of Pb content was established in horse meat samples from the Bukhar-Zhyrau district at 0.71 ± 0.013 mg/kg (MPCs 0.5 mg/kg); in lamb samples from the same district, its concentration was 1.11 ± 0.046 mg/kg (2.2 times higher than the norm), and in horse meat samples from the Aktogay district, it reached 1.94 ± 0.0024 mg/kg (4 times higher than the norm). The Cd content in samples from the Bukhar-Zhyrau district was 0.11 ± 0.64 mg/kg in lamb and 0.216 ± 2.26 mg/kg in horse meat, which exceeds the MPCs by 2.2 and 4.3 times, respectively. The Cu content in beef from the Bukhar-Zhyrau district was 5.07 ± 0.024 mg/kg, and that in the Aktogay district 52.95 ± 8.26 mg/kg, with an MPCs of 5 mg/kg (exceeding it by 10.6 times). The Zn content in horse meat samples from the Aktogay district was 155.3 ± 2.26 mg/kg, which exceeds the MPCs by 2.2 times; it was within the permissible range in the remaining meat samples.

**Figure 1:**
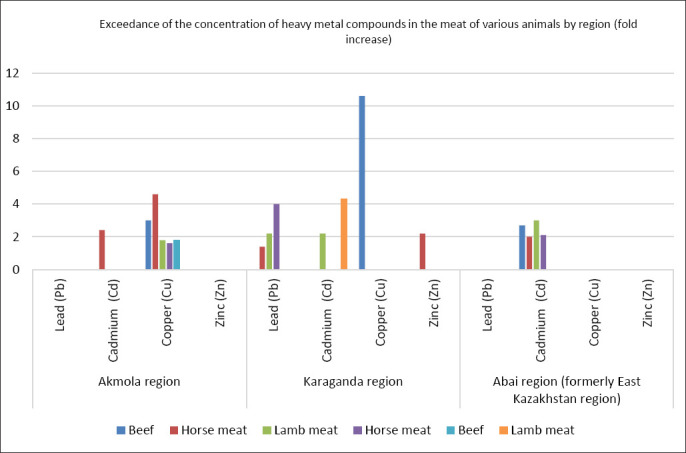
Exceedance of the concentration of heavy metal compounds in the meat of various animals by region (fold increase).

The concentrations of Pb, Cu, and Zn in the studied meat samples in the Abai region (formerly the East Kazakhstan region) did not exceed the MPCs. However, an increased Cd content was noted in all samples from the Abai district: in beef it was 0.135 ± 0.035 mg/kg (2.7 times higher than the norm), in horse meat 0.1 ± 0.075 mg/kg (2 times higher than the norm), and in lamb 0.14 ± 0.035 mg/kg (3 times higher than the norm). In the Ayagoz district, an MPCs exceedance for Cd was established only in horse meat samples, where its content was 0.105 ± 0.015 mg/kg. Figure 1 shows a general overview of MPCs exceedances of toxic elements in the meat of various animals by region.

Thus, in the Akmola region, MPCs exceeded for Cu in beef, horse meat, and lamb meat from the Zerenda and Akkol districts, as well as for Cd in individual samples of horse meat from the Shortandy district. Meat from animals in the Karaganda region was found to be more susceptible to contamination with toxic elements, with exceedances of permissible concentrations for Pb, Cd, Cu, and Zn recorded in almost all meat types. In the study of meat samples from the Abai region (formerly the East Kazakhstan region), an exceedance was established only for Cd.

### Poultry meat samples

The results of the analysis of poultry meat contamination with toxic elements are presented in Table 2. Cadmium was detected in chicken meat from the Zerenda district in the Akmola region at a concentration of 0.21 ± 0.0032 mg/kg, with a standard of 0.05 mg/kg (an exceedance by 4.2 times). Cadmium was absent in most samples in the Karaganda and Abai regions, and no exceedances of the MPCs were recorded.

**Table 2:** Content of toxic elements in poultry meat.

Regions	Type of meat	Lead (mg/kg)	Cadmium (mg/kg)	Copper (mg/kg)	Zinc (mg/kg)
Akmola region					
Zerenda	Chicken meat, n = 6	0.014 ± 0.00012	0.21 ± 0.0032	0.21 ± 0.0031	2.7 ± 0.36
	Duck meat, goose meat, n = 6	0.00012 ± 0.000	n/d	n/d	6.2 ± 0.24
Akkol	Chicken meat, n = 6	0.12 ± 0.04	0.01 ± 0.003	0.09 ± 0.005	n/d
	Duck meat, goose meat, n = 6	n/d	n/d	n/d	n/d
Bulandy	Chicken meat, n = 6	0.11 ± 0.04	n/d	0.12 ± 0.003	n/d
	Duck meat, goose meat, n = 6	n/d	n/d	n/d	n/d
Karaganda region					
Bukhar-Zhyrau	Chicken meat, n = 6	n/d	n/d	n/d	4.2 ± 0.81
	Duck meat, goose meat, n = 6	n/d	n/d	n/d	n/d
Abai	Chicken meat, n = 6	0.11 ± 0.04	0.011 ± 0.04	0.013 ± 0.005	n/d
	Duck meat, goose meat, n = 6	n/d	n/d	n/d	n/d
Aktogay	Chicken meat, n = 6	n/d	0.016 ± 0.06	n/d	14.6 ± 2.26
	Duck meat, goose meat, n = 6	n/d	n/d	n/d	16.8 ± 4.34
Abai region (formerly the East Kazakhstan region)					
Abai	Chicken meat, n = 6	0.212 ± 0.047	0.025 ± 0.050	1.705 ± 0.56	5.88 ± 0.93
	Duck meat, goose meat, n = 6	n/d	n/d	n/d	n/d
Ayagoz	Chicken meat, n = 6	0.108 ± 0.026	0.038 ± 0.013	2.15 ± 0.54	4.17 ± 0.59
	Duck meat, goose meat, n = 6	n/d	n/d	n/d	n/d
Aksuat	Chicken meat, n = 6	0.122 ± 0.042	0.028 ± 0.015	1.61 ± 0.40	4.10 ± 0.30
	Duck meat, goose meat, n = 6	n/d	n/d	n/d	n/d
MPCs		0.5	0.05	5	70

Lead was detected mainly in chicken meat from all regions; it was absent in duck and goose meat, with no exceedances of aximum permissible concentrations.

Copper and zinc were detected in all chicken meat samples from the Akmola and Abai regions, and in the Karaganda region in chicken meat from the Abai district; the values were within the permissible limits.

Thus, poultry meat from all the studied regions meets the sanitary and hygienic standards for toxic elements, except for individual chicken meat samples from the Zerenda district of the Akmola region in terms of Cd content. In general, the products are considered safe for consumption.

### Analysis of milk and dairy products

The degree of contamination of milk and dairy products (cottage cheese and homemade cheeses) with toxic elements and radionuclides was studied (Table 3).

**Table 3:** Toxic element content in milk and dairy products.

Regions	Milk product	Lead (mg/kg)	Cadmium (mg/kg)	Copper (mg/kg)	Zinc (mg/kg)
Akmola region					
Zerenda	Whole milk, n = 6	0.048 ± 0.019	n/d	1.07 ± 0.041	n/d
	Cottage cheese, homemade cheese, n = 6	0.062 ± 0.024	0.058 ± 0.023	0.315 ± 0.041	0.2 ± 0.12
Akkol	Whole milk, n = 6	0.085 ± 0.33	n/d	0.0082 ± 0.0032	n/d
	Cottage cheese, homemade cheese, n = 6	0.024 ± 0.004	0.061 ± 0.0013	0.216 ± 0.0026	0.038 ± 0.022
Bulandy	Whole milk, n = 6	0.00067 ± 0.0002	n/d	0.075 ± 0.0032	1.4 ± 0.5
	Cottage cheese, homemade cheese, n = 6	0.0023 ± 0.00026	n/d	n/d	n/d
Karaganda region					
Bukhar-Zhyrau	Whole milk, n = 6	0.038 ± 0.015	n/d	n/d	n/d
	Cottage cheese, homemade cheese, n = 6	0.112 ± 0.0010	n/d	0.178 ± 0.043	n/d
Abai	Whole milk, n = 6	0.0054 ± 0.0021	0.0062 ± 0.0021	n/d	n/d
	Cottage cheese, homemade cheese, n = 6	0.0025 ± 0.0010	0.034 ± 0.013	0.5 ± 0.23	0.11 ± 0.04
Aktogay	Whole milk, n = 6	0.0021 ± 0.0021	0.072 ± 0.0021	n/d	n/d
	Cottage cheese, homemade cheese, n = 6	0.011 ± 0.016	n/d	n/d	n/d
Abai region (formerly the East Kazakhstan region)					
Abai	Whole milk, n = 6	0.272 ± 0.179	0.020 ± 0.007	0.346 ± 0.261	4.30 ± 0.446
Ayagoz	Whole milk, n = 6	0.178 ± 0.073	0.022 ± 0.008	0.374 ± 0.217	4.37 ± 0.529
Aksuat	Whole milk, n = 6	0.134 ± 0.043	0.024 ± 0.009	0.180 ± 0.131	4.10 ± 0.300
MPCs		0.1	0.03	1.0	5.0

During the analysis of milk and dairy products from the Akmola region, it was established exceedance of Cd content was recorded in cottage cheese samples from the Zerenda district at 0.058 ± 0.023 mg/kg (MPCs ≤ 0.03 mg/kg) and in cottage cheese from the Akkol district at 0.061 ± 0.0013 mg/kg, as well as a slight exceedance of Cu content in milk samples from the Zerenda district at 1.07 ± 0.041 mg/kg (MPCs ≤ 0.1 mg/kg). In the Karaganda region, a slight exceedance of the MPCs for Pb was recorded in cottage cheese from the Bukhar-Zhyrau district at 0.112 ± 0.0010 mg/kg (MPCs ≤ 0.1 mg/kg), as well as for Cd in cottage cheese from the Abai district (0.034 ± 0.013 mg/kg) and in whole milk from the Aktogay district (0.072 ± 0.0021 mg/kg) with an MPCs of no more than 0.03 mg/kg. In the Abai region, MPCs exceeded for Pb were recorded in all samples of whole milk: Aksuat district, 0.134 ± 0.043 mg/kg; Ayagoz district, 0.178 ± 0.073 mg/kg; and Abai district, 0.272 ± 0.179 mg/kg (MPCs ≤ 0.1 mg/kg). No exceedances of the permissible limits for Cu and Zn were detected in milk and dairy product samples in the Karaganda and Abai regions.

Thus, the following MPCs exceedances were identified: in the Karaganda region, Pb was present in cottage cheese, Cd in milk, and cottage cheese; in the Abai region, Pb was present in all samples of whole milk; in the Akmola region, Cu was present in individual samples of whole milk, and Cd was present in cottage cheese samples.

### Analysis of freshwater fish

The contamination of fish with toxic elements in natural water bodies of the Akmola and Karaganda regions was investigated. The results are presented in Table 4.

**Table 4:** Contamination of fish with toxic elements in the Akmola and Karaganda regions.

Regions	Fish species	Cadmium (mg/kg)	Lead (mg/kg)	Arsenic (mg/kg)	Mercury (mg/kg)
Akmola region					
Burabai	Peled, n = 11	n/d	0.025 ± 0.00013	0.0347 ± 0.001	0.022 ± 0.0012
	Ripus, n = 9	0.025 ± 0.006	0.022 ± 0.006	0.036 ± 0.01	n/d
	Carassius spp., n = 6	0.037 ± 0.009	0.030 ± 0.008	0.024 ± 0.006	n/d
	Crucian carp, n = 12	0.0046 ± 0.001	0.0083 ± 0.0001	0.035 ± 0.0012	0.0206 ± 0.0002
Zerenda	Crucian carp, n = 10	n/d	0.075 ± 0.007	0.020 ± 0.004	n/d
	Rutilus rutilus, n = 7	0.085 ± 0.002	0.033 ± 0.006	0.043 ± 0.002	n/d
Shortandy	Crucian carp, n = 12	0.075 ± 0.018	0.0272 ± 0.028	0.032 ± 0.0012	0.22 ± 0.0021
Korgalzhin	Crucian carp, n = 12	n/d	n/d	n/d	n/d
Karaganda region					
Bukhar-Zhyrau	Tench, n = 15	n/d	0.0394 ± 0.002	n/d	n/d
	Pike-perch, n = 13	n/d	0.06 ± 0.0002	n/d	n/d
	Sander lucioperca, n = 12	n/d	0.0014 ± 0.000	n/d	0.03 ± 0.0003
Nura	Crucian carp, n = 10	n/d	0.059 ± 0.0001	n/d	0.02 ± 0.0001
Abai	Crucian carp, n = 15	n/d	0.05 ± 0.003	n/d	n/d
MPCs		0.2	1.0	1.0	0.3

Toxic elements were detected in fish samples at low levels that did not exceed the MPCs, and elements such as Cd and Hg were absent in most cases. Residual Pb levels were detected in the majority of the analyzed fish samples from water bodies of the Akmola region, ranging from 0.0083 ± 0.00012 mg/kg to 0.075 ± 0.007 mg/kg, while As concentrations ranged from 0.020 ± 0.007 mg/kg to 0.043 ± 0.002 mg/kg. In both cases, the MPCs were ≤1.0 mg/kg.

In the Karaganda region, Pb concentrations in fish muscle tissue ranged from 0.0394 ± 0.002 mg/kg to 0.06 ± 0.0002 mg/kg, with an MPCs of ≤1.0 mg/kg. Mercury was detected only in fish from two water bodies, at levels of 0.02 ± 0.0001 mg/kg to 0.03 ± 0.0003 mg/kg, which is below the MPCs of ≤0.3 mg/kg. Cadmium and arsenic were not detected in the muscle tissue of fish.

Therefore, freshwater fish from the Akmola and Karaganda regions can be considered safe with respect to toxic element content.

### Analysis of residual radionuclide levels in meat, milk and dairy products, and fish

The results of radionuclide concentration measurements in meat from different animal species are presented in Table 5.

**Table 5:** Radionuclide content in the meat of farm animals.

Regions	Type of meat	Cesium 137 (Bq/kg)	Strontium 90 (Bq/kg)
Akmola region			
Zerenda	Beef, n = 8	12.16 ± 0.062	n/d
	Horse meat, n = 6	16.52 ± 0.026	2.62 ± 0.038
	Lamb meat, n = 6	8.26 ± 0.002	n/d
Akkol	Beef, n = 8	12.62 ± 0.004	1.76 ± 0.022
	Horse meat, n = 6	10.12 ± 0.006	n/d
	Lamb meat, n = 6	2.83 ± 0.004	n/d
Shortandy	Beef, n = 8	12.34 ± 0.003	n/d
	Horse meat, n = 6	18.06 ± 0.026	n/d
	Lamb meat, n = 6	1.73 ± 0.008	n/d
Karaganda region			
Bukhar-Zhyrau	Beef, n = 6	1.18 ± 0.021	n/d
	Horse meat, n = 6	6.51 ± 0.012	n/d
	Lamb meat, n = 6	3.36 ± 0.002	0.38 ± 0.026
Abai	Beef, n = 6	2.08 ± 0.023	n/d
	Horse meat, n = 6	1.92 ± 0.003	n/d
	Lamb meat, n = 6	1.47 ± 0.0024	n/d
Aktogay	Beef, n = 6	2.34 ± 0.0013	n/d
	Horse meat, n = 6	3.06 ± 0.026	n/d
	Lamb meat, n = 6	2.73 ± 0.008	n/d
Abai region (formerly the East Kazakhstan region)			
Abai	Beef, n = 6	18.16 ± 0.0002	0.94 ± 0.04
	Horse meat, n = 6	15.53 ± 0.0001	0.80 ± 0.15
	Lamb meat, n = 6	17.34 ± 0.0003	0.90 ± 0.05
Ayagoz	Beef, n = 6	12.07 ± 0.0005	0.62 ± 0.041
	Horse meat, n = 6	10.95 ± 0.0003	0.57 ± 0.21
	Lamb meat, n = 6	9.41 ± 0.00004	0.49 ± 0.031
Aksuat	Beef, n = 6	5.54 ± 0.00013	0.29 ± 0.24
	Horse meat, n = 6	4.07 ± 0.00021	0.21 ± 0.42
	Lamb meat, n = 6	4.88 ± 0.00018	0.25 ± 0.13
MPCs		200	100

As shown in Table 5, radionuclide concentrations in the analyzed samples were substantially below the permissible limits. In samples from the Akmola region, 137 Cs levels ranged from 1.73 ± 0.008 Bq/kg to 18.06 ± 0.02 Bq/kg; in the Karaganda region, from 1.18 ± 0.021 Bq/kg to 6.51 ± 0.012 Bq/kg; and in the Abai region, from 4.07 ± 0.00021 Bq/kg to 18.16 ± 0.0002 Bq/kg, with an MPCs of ≤200 Bq/kg.

Strontium-90 (90Sr) was detected in samples from the Akmola region in two cases only: horse meat (2.62 ± 0.038 Bq/kg) and beef (1.76 ± 0.022 Bq/kg). In the Karaganda region, it was detected only in mutton (0.38 ± 0.026 Bq/kg). Strontium-90 (90Sr) was detected in all samples in the Abai region, ranging from 0.21 ± 0.42 Bq/kg to 0.94 ± 0.04 Bq/kg.

Although no MPCs exceedances were observed, the highest and lowest radionuclide concentrations were recorded in meat products from the Abai region and Karaganda region, respectively. A similar pattern was observed for meat and dairy products.

Radionuclides were detected in most fish samples analyzed for residual radionuclides (86.3% of the total number of samples); however, no MPCs exceedances were identified (Table 6).

**Table 6:** Radionuclide content in fish meat from water bodies of Central Kazakhstan.

Regions	Type of fish	Cesium 137 (Bq/kg)	Strontium 90 (Bq/kg)
Akmola region			
Burabay	Peled, n = 11	8.76 ± 0.64	11.33 ± 0.02
	Ripus, n = 9	9.03 ± 1.12	13.30 ± 0.60
	Crucian carp, n = 6	15.30 ± 0.12	4.8 ± 0.06
	Crucian carp, n = 12	9.03 ± 0.12	11.80 ± 0.002
Zerenda	Crucian carp, n = 10	16.6 ± 0.26	10.6 ± 0.00
	Roach, n = 7	11.4 ± 0.6	6.50 ± 0.2
Shortandy	Crucian carp, tench, n = 12	26.13 ± 0.02	10.96 ± 0.26
Korgaldzhin	Crucian carp, n = 12	n/d	9.20 ± 0.80
Karaganda region			
Nura	Crucian carp, n = 10	n/d	1.07 ± 0.0012
Bukhar-Zhyrau	Tench, n = 15	2.07 ± 0.021	10.24 ± 0.03
	Perch, n = 13	2.3 ± 0.001	n/d
	Pike-perch, n = 12	23.64 ± 0.016	5.9 ± 0.023
Abai	Crucian carp, n = 15	n/d	1.02 ± 0.02
MPCs		130	100

Residual 137 Cs levels in fish samples from the Akmola region ranged from 8.76 ± 0.64 Bq/kg (ripus) to 26.13 ± 0.02 Bq/kg (crucian carp). In the Karaganda region, 137 Cs concentrations ranged from 2.07 ± 0.021 Bq/kg (tench) to 23.64 ± 0.016 Bq/kg (pike-perch), with an MPCs of 130 Bq/kg. The 90Sr content in fish from the Akmola region water bodies ranged from 6.50 ± 0.2 Bq/kg (roach) to 13.3 ± 0.6 Bq/kg (ripus). In the Karaganda region, 90Sr levels ranged from 1.02 ± 0.02 Bq/kg to 10.24 ± 0.03 Bq/kg (tench), with an MPCs of ≤100 Bq/kg. No statistically significant differences were observed between regions or fish species (*p* > 0.05).

The residual levels of cesium and strontium radionuclides in meat from various animal species, poultry, milk, and freshwater fish muscle tissue did not exceed the permissible limits established by sanitary and epidemiological requirements, indicating radiological safety.

## DISCUSSION

### Regional patterns of toxic element contamination

The present comprehensive study revealed a region-specific distribution of toxic element contamination in livestock products and fish from the Akmola, Karaganda, and Abai (formerly East Kazakhstan) regions of the Republic of Kazakhstan. The most pronounced exceedances of MPCs were recorded in meat samples from the Karaganda region, where elevated levels of Pb, Cd, Cu, and Zn were detected in nearly all types of meat (beef, horse meat, and mutton). In the Akmola region (Zerendinsky and Akkol districts), exceedances were mainly associated with Cu in beef, horse meat, and mutton, as well as Cd in individual horse meat samples from the Shortandinsky district. In the Abai region, Cd was predominantly involved in the exceedances. In poultry meat (chicken) from the Akmola region, cadmium levels exceeded the regulatory limit by 4.2-fold, whereas Pb, Cu, and Zn levels remained within permissible ranges.

### Industrial and environmental determinants

These findings are consistent with previous reports indicating a high technogenic burden in the Karaganda and Abai regions, where mining and metallurgical industries have historically been well developed [37]. The predominance of Cu exceedances may be explained by combined anthropogenic and agrotechnical contributions, including emissions from mining and metal-processing industries, as well as the use of Cu-containing pesticides, fertilizers, and soil amendments [38]. Agrochemical data indicate that mineral fertilizer application increases soil Cu content by 8.3%, Zn by 24%, Cd by 26%, and Pb by 7.3%, which enhances the “soil → feed → animals → food products” pathway [39]. An additional factor is contamination of surface waters (e.g., the Irtysh River in the Abai region), where Cd, Hg, and Zn concentrations exceed the hydrosphere Clarke values by 1.7–7-fold, thereby increasing bioavailability during irrigation and livestock watering [40].

### Poultry contamination and feeding-related factors

In poultry meat, contamination levels were generally lower (except for Cd in samples from the Akmola region), which may be attributed to feeding practices based primarily on grain compound feeds, which tend to exhibit lower accumulation than pasture-based diets in ruminants. Nevertheless, grain quality dependence (including crops produced under intensive fertilizer application) remains an important factor [41]. Modern poultry production systems and environmental contamination may increase the risk of toxic contaminants entering the human body through food consumption [42].

### Comparison with international meat contamination data

According to data from the United States Department of Agriculture Food Safety and Inspection Service (USDA FSIS) (USA, 2017–2022; more than 13,000 meat and poultry samples), studies from China (1,066 fresh meat samples), and global meta-analyses of red meat, As and Hg are generally below Codex Alimentarius limits, whereas Pb and Cd often exceed permissible levels, particularly in Asia and Africa. The reported mean values were 1.02 mg/kg Pb and 0.23 mg/kg Cd in Asia and 0.97 mg/kg Pb and 0.085 mg/kg Cd in Africa [43–45]. Therefore, red meat consumption may pose a substantial health risk in Asia and Africa, whereas the risk appears minimal in Europe and North America; however, continuous monitoring is still required [43–45].

### Milk and dairy product contamination pathways

Concentrations of Pb, Cd, Cu, and other metals in milk and dairy products were generally below MPCs, although elevated Pb and Cd were observed in certain regions, likely due to prevailing wind patterns and industrial emissions. Pb and Cd may accumulate in plants and soil as a result of vehicle exhaust, the application of mineral fertilizers (superphosphate, potassium phosphate, nitrate), and fungicides [46]. The concentrations of these metals in milk differ substantially between cattle grazing on contaminated and uncontaminated soils. Processing equipment and packaging materials are additional sources of Pb, and metal levels may increase with higher storage temperatures and prolonged processing times [47, 48]. Cu may enter milk not only from environmental sources but also via compound feeds, fertilized grains, and premixes containing Cu as a trace element to enhance animal productivity, particularly when supplementation is inadequately regulated [49].

### Global dairy contamination evidence

International studies (2015–2025) have further confirmed regional variability. For example, the reported concentrations of Cd, Hg, and Pb in hard cheese from Italy were 0.004, 0.08, and 0.13 mg/kg, respectively. In Mexico, Pb in Ranchero cheese reached 0.11 mg/kg with exceedances in some samples. In Peru, Cd and Pb ranged from 0.018 to 0.178 mg/kg and from 0.217 to 0.58 mg/kg, respectively, and frequently exceeded limits in mining areas. In Iran, Pb in raw milk reached 0.53 mg/kg, and in Bangladesh, Pb exceeded regulatory limits in 13%–25% of powdered milk samples [50, 51]. A meta-analysis of 66 studies worldwide assessing As, Hg, Pb, and Cd reported that concentrations are generally below permissible limits, although elevated Pb and Cd occur in specific regions, indicating the need for continuous monitoring [52].

### Freshwater fish contamination and environmental context

Residual levels of toxic elements (heavy metals) were detected in freshwater fish from the Akmola, Karaganda, and Abai regions of Kazakhstan; however, the concentrations did not exceed the regulatory limits. These findings are consistent with published evidence showing that heavy metal contamination of water bodies is strongly associated with technogenic load, industrial emissions, and hydrological characteristics [26]. In contrast to heavily polluted “hot spots” in Central Asia (e.g., the Syr Darya Basin and the Small Aral Sea, where As concentrations in water exceed WHO recommendations by 2–7-fold and Cd and Pb exceed standards by 1.5–5-fold), as well as the Ili River (Cd 1.7–28.7 µg/L and Pb 0.2–87.0 µg/L, exceeding WHO standards by more than five-fold at certain sites), the investigated water bodies demonstrated a more favorable environmental situation [53]. Elevated Zn levels in the Irtysh River (Eastern Kazakhstan) have been reported [54], but without substantial transfer to edible fish tissues. Similarly, in the Tobol–Turgay and Ishim basins (Northern Kazakhstan), heavy metal concentrations in fish are generally below permissible levels, except in localized areas affected by industrial activities [55].

### International comparisons of fish contamination

Comparisons with international and regional data confirm a low risk: in the most recent studies on freshwater fish in Central Asia (2020–2025), concentrations of Hg, Cd, Pb, and As in fish muscle tissue remain below the maximum allowable levels established by Codex Alimentarius and national regulations. Although Pb may reach 0.5–3 mg/kg and Cd 0.1–1.5 mg/kg in certain tissues in heavily polluted rivers (e.g., in China, India, Pakistan, and Iraq), and Hg in predatory species may reach 0.6–1.0 mg/kg (exceeding MPCs by 2–3-fold) [56, 57], both farmed and wild freshwater fish generally show lower levels in Kazakhstan, particularly in less industrialized areas [58].

### Monitoring implications and food safety considerations

Overall, the obtained results highlight the relative safety of freshwater fish from the studied regions with respect to heavy metals and radionuclides (^137^Cs and ^90^Sr were also within regulatory limits). Nevertheless, considering the transboundary nature of major rivers (Irtysh, Ishim, and Tobol) and ongoing technogenic pressure in Central Asia, the following recommendations are proposed: regular monitoring of heavy metals in water, bottom sediments, and fish (especially in industrial impact zones and downstream of pollution sources) [59], preference for fish obtained from certified producers or less contaminated areas when consumed frequently [60], and further studies including quantitative assessment, trophic-level bioaccumulation analysis, and health risk estimation for local populations.

### Radionuclide contamination assessment

With respect to radionuclides (^137^Cs and ^90^Sr), no exceedances of maximum permissible levels were observed in meat, milk, dairy products, or fish. Concentrations were low or below detection limits in most regions, with localized increases near the former SNTS; however, regulatory thresholds were not exceeded. This is consistent with post-nuclear monitoring reports, where the expected levels of ^137^Cs in meat during grazing are 22–44 Bq/kg and those of ^90^Sr are 1.1–8.9 Bq/kg [35]. The levels of artificial radionuclides in milk and dairy products are substantially below the limits established by the International Atomic Energy Agency (IAEA), WHO, and Codex Alimentarius [61–65].

### One Health perspective and policy implications

The results of this study emphasize the critical importance of the One Health approach in ensuring environmental and food safety. The observed patterns indicate the need to strengthen governmental monitoring of heavy metals and radionuclides in environmental matrixes (soil and water), feed resources, and livestock products, particularly in Kazakhstan’s industrial and post-nuclear regions.

## CONCLUSION

This comprehensive monitoring study demonstrated a clear region-specific pattern of contamination of livestock products and freshwater fish in industrially influenced regions of Kazakhstan. Exceedances of MPCs for toxic elements were detected mainly in meat and dairy products, whereas fish samples showed relatively low contamination levels. The Karaganda region exhibited the highest environmental burden, with elevated concentrations of Pb, Cd, Cu, and Zn recorded in several meat samples. In the Akmola region, exceedances were primarily associated with Cu in meat and Cd in individual poultry and livestock samples. In the Abai region, contamination was mainly linked to Cd in meat and Pb in whole milk. Despite these localized exceedances, radionuclide concentrations (^137^Cs and ^90^Sr) in meat, dairy products, and fish remained substantially below regulatory thresholds in all studied regions.

From a public health perspective, these findings confirm that most livestock products and freshwater fish from the investigated territories are generally safe for consumption; however, the presence of localized contamination hotspots indicates a potential risk of chronic exposure for populations regularly consuming locally produced food products. The results highlight the importance of strengthening routine monitoring systems for toxic elements in food products, particularly in regions affected by mining, metallurgy, and historical industrial activities. Enhanced control of environmental emissions, feed quality, irrigation water, and agricultural inputs may help reduce the transfer of contaminants along the “soil → feed → animal → food product” pathway.

A major strength of this study is its integrated regional approach, combining analysis of multiple food matrixes (meat from different animal species, poultry, milk and dairy products, and freshwater fish) across several industrially influenced regions. The use of standardized analytical methods and inclusion of both toxic elements and radionuclides provide a comprehensive assessment of chemical and radiological safety. The relatively large number of samples collected and the inclusion of products from both farms and retail markets further enhance the representativeness of the results.

Nevertheless, several limitations should be acknowledged. The cross-sectional design reflects contamination levels during a specific monitoring period and does not capture seasonal or long-term temporal variability. Environmental matrixes such as soil, water, and feed were not analyzed simultaneously, which limits direct identification of contamination sources and pathways. In addition, the study did not include a quantitative dietary exposure assessment or human health risk modeling, which would allow estimation of population-level risks associated with long-term consumption of contaminated products.

Future research should therefore focus on longitudinal monitoring to evaluate temporal trends in contamination, integrated ecosystem studies linking soil, water, feed, and animal products, and comprehensive health risk assessments incorporating consumption patterns of local populations. Further investigations into bioaccumulation mechanisms, trophic transfer, and spatial modeling of contamination hotspots would also improve environmental risk management and food safety planning.

In conclusion, although most tested livestock products and freshwater fish from the studied regions meet established safety standards, the detection of localized exceedances of toxic elements underscores the continued need for systematic environmental and food monitoring. Strengthening surveillance programs, improving industrial emission control, and implementing preventive agricultural practices will be essential to ensure long-term food safety, protect public health, and support sustainable agricultural development in Kazakhstan’s industrial and post-industrial regions.

## DATA AVAILABILITY

The supplementary data can be made available from the corresponding author upon request.

## AUTHORS’ CONTRIBUTIONS

AZh: Conceptualization and manuscript drafting. MR, SSh, and SA: Methodology, statistical analyses, and critical revision. ZG and SZh: Experiments and data collection. BA: Interpretation of results and drafted and revised the manuscript. All authors have read and approved the final version of the manuscript.
